# The Association of Salivary Flow Rate and Sleep Quality among Head and Neck Cancer Survivors after Radiotherapy

**DOI:** 10.1186/s12903-024-03977-5

**Published:** 2024-02-19

**Authors:** Yen-Wen Shen, Wen-Chen Wang, Nan-Chin Lin, Valendriyani Ningrum, Tzong-Ming Shieh, Yin-Hwa Shih

**Affiliations:** 1 School of Dentistry, China Medical University, Taichung, 40402 Taiwan; 2 Department of Dentistry, China Medical University Hospital, Taichung City, 404332 Taiwan; 3 Department of Oral Pathology, College of Dental Medicine, Kaohsiung Medical University, Kaohsiung, 80708 Taiwan; 4 Division of Oral Pathology & Maxillofacial Radiology, Kaohsiung Medical University Hospital, Kaohsiung, 80708 Taiwan; 5 Oral & Maxillofacial Imaging Center, College of Dental Medicine, Kaohsiung Medical University, Kaohsiung, 80708 Taiwan; 6 Department of Oral and Maxillofacial Surgery, Show Chwan Memorial Hospital, Changhua, 500009 Taiwan; 7 School of Dentistry, Baiturrahmah University, By Pass Km 15 Aie Pacah, Padang, 25586 Indonesia; 8 Department of Healthcare Administration, Asia University, Taichung, 41354 Taiwan

**Keywords:** Pittsburgh sleep quality index, Salivary flow, DMFT, Radiotherapy, Head and neck cancer

## Abstract

**Background:**

Head and neck cancer survivors suffer from xerostomia and sleep disturbances after radiotherapy, both of which affect their quality of life. This study aimed to explore the role of salivary flow in the oral health and sleep quality of head and neck cancer survivors.

**Methods:**

We recruited 120 head and neck cancer survivors who were experiencing symptoms of dry mouth or sleep disturbances post-radiotherapy from a dental clinic. We gathered their socio-demographic and clinical data, measured their salivary flow rate, and recorded their dry mouth score using the summated xerostomia inventory. Additionally, a dentist collected the DMFT (Decayed, Missing, and Filled Teeth) index. The Pittsburgh Sleep Quality Index was employed to assess their sleep quality.

**Results:**

In this study, xerostomia was observed in nearly 80% of the cancer survivors. The concurrent prevalence of sleep disturbance and xerostomia was at 55%. After five years post-radiotherapy, there was a significant improvement observed in both the quality of sleep (*p* = 0.03) and the stimulated salivary flow rate (*p* = 0.04). Additionally, these improvements were noted to have commenced from the third year onwards. A significant association was found between stimulated salivary flow and dry mouth scores with poor sleep quality (p <  0.05).

**Conclusions:**

We recommend that dental professionals prioritize managing both dental and mental health issues equally for head and neck cancer survivors who have undergone radiotherapy within the past 3 years.

**Supplementary Information:**

The online version contains supplementary material available at 10.1186/s12903-024-03977-5.

## Background

Head and neck cancer is the seventh most common cancer globally [1] and the fourth most common cancer in Taiwanese men. In Asia, the risk factors contributing to head and neck cancers include alcohol consumption, betel nut chewing, cigarette smoking, and viral infections [2]. Approximately 90% of head and neck cancers are squamous cell carcinoma [3]. Head and neck cancers are a group of malignancies that arise from the squamous cell lining of the larynx, throat, lips, mouth, nose, and salivary glands.

Head and neck cancers can be cured if detected early. Treatment methods include surgery, radiation therapy, and medication therapy [4]. The treatment options for head and neck cancer depend on the type and stage of the cancer. Surgery aims to remove cancerous tissues; however, the side effects include facial appearance changes and oral function limitations. Radiotherapy aims to cure cancer. Side effects include loss of taste, dry mouth, pain, mouth sores, and hypothyroidism. Medications aim to destroy cancer cells, either systematically or locally. Side effects commonly occur in the skin and gastrointestinal tract.

After the cancer is eliminated, preserving the function of nearby nerves and tissues in the oral cavity is important for patients’ quality of life. Side effects of radiotherapy to the head and neck region may usually occur toward the end of the course of treatment and during the first few weeks after the completion of treatment. A long course of treatment may cause more adverse effects than a short course. Problems include acute toxicity that induces oral mucosa and throat inflammation, loss of taste, and chronic toxicity to hearing function, thyroid function, and salivary secretions [5]. Saliva contains numerous proteins that maintain several functions in the oral cavity. Thus, patients who receive radiotherapy and have dry mouth are more susceptible to dental diseases and a worsen sleep quality [6–8]. In contrast, individuals with poor sleep quality have a higher prevalence of morning hyposalivation and xerostomia [9]. One symptom is exacerbated when the other symptom worsens.

Cancer survivors must live an everyday life after receiving cancer treatment. Supportive care for dry mouth and sleep quality from dentists is important for maintaining their quality of life. Head and neck cancer survivors usually visit a dentist and have their teeth checked every 1 or 2 months for at least 6 months after the end of radiation treatment. The dentist examines changes in their mouth and provides proper management to support them in completing the treatment course [10, 11]. However, the dentist can not adress the xerostomia-related sleep disturbance problem simutaneously.

Based on the literature review, the exact time point and duration of sleep intervention at the bedside and an objective marker to predict oral health and sleep quality among head and neck cancer survivors are lacking. This study aimed to investigate the relationship between salivary flow, oral health issues, and sleep disturbances in survivors of head and neck cancer who have undergone radiotherapy, as well as to assess the necessity of referring patients to a sleep clinic.

## Methods

### Participant enrollment

This study enrolled patients with head and neck cancer who had received concurrent radiotherapy and had a dental visit at the Department of Oral Pathology, College of Dental Medicine, Kaohsiung Medical University. In total, 120 participants were enrolled in this study. The sample size was determined to be five times that of the Pittsburgh Sleep Quality Index (PSQI) items. We included adults with dry mouth symptoms after cancer therapy and excluded the confounding factors that may have resulted in dry mouth in adults with a history of smoking, immune disease, psychiatric disease, pregnancy, or taking medicines that led to dry mouth effects. The oral health status was examined by dentists. Social demographics, salivary flow rates, dry mouth scores, and sleep quality data were collected by a research assistant.

### Collection of the saliva

Saliva was collected immediately after the oral examination. The saliva flow rate test was performed in a narrow time between 9:30 am and 12:30 pm to avoid the circadian effect [12] and obtain an accurate definition of hyposalivation. The participants were instructed to refrain from eating, smoking, and drinking coffee and tea for 1.5 hr. before saliva collection. The consumption of tap water was also permitted. The participants were asked to relax for a few minutes before saliva collection. Saliva was collected using the spitting method. The whole saliva was collected within 5 min, and every 30 s, the participants spit all the saliva available in the mouth. After a 2-minute break, the stimulated whole saliva was collected within 5 min using a tasteless piece of Parafilm® M (Merck, Darmstadt, Germany) (5X5 cm 0.3 g). Every 30 s, the participants spit all the saliva available in their mouths. The whole-saliva flow rate was determined by gravitation, assuming that 1 g of saliva was equivalent to 1 mL. The hyposalivation cut-off values were 0.1 ml/min for unstimulated saliva and 0.5 ml/min for chewing-stimulated saliva. We defined a patient as having xerostomia when the salivary flow rate met the criteria for hyposalivation.

$$\textrm{Salivary}\ \textrm{Flow}\ \textrm{Rate}=\frac{\left(\textrm{post} \textrm{weight}\ \textrm{measurement} - \textrm{pre}\textrm{weight}\ \textrm{measurement}\right)}{\textrm{collection}\ \textrm{period}}=\textrm{g}/\min =\textrm{mL}/\min$$

### Collection of the questionnaire data

The demographic data of age, sex, educational level, major diagnosis of cancer type, years after radiotherapy treatment, and DMFT were collected from the electronic medical history records. Sleep disorders were assessed by using the PSQI. The Chinese version had a Cronbach’s alpha of 0.82. A total score of 5 or higher indicates sleep disturbance, with higher scores indicating severe disturbance. We evaluated dry mouth scores using the Chinese version of the Summated Xerostomia Inventory [13]. Cronbach’s alpha was 0.81 in the present study. Summated Xerostomia Inventory had five items (my mouth feels dry when eating a meal; my mouth feels dry; I have difficulty in eating dry food; I have difficulties swallowing certain foods; and my lips feel dry), with a respondent asked to choose one of three response options(“Never” scoring 1; “Cccasionally” scoring 2; and “Often” scoring 5). The total score is the sum of the five individual item scores (range 5–15). Higher scores indicated greater severity of dry mouth symptoms. The DMFT (Decayed, Missing, and Filled Teeth) index was determined by a clinical dentist. We use the average value of 12 as a cut-off point. A higher DMFT index indicates a higher incidence of dental caries.

### Statistics

Statistical analysis was conducted using the SPSS software (IBM, Armonk, New York, USA), and continuous variables were tested for non-normality using the Kolmogorov test, with *p* <  0.05. This study used nonparametric statistical methods (Kruskal-Wallis test and Mann-Whitney U test) to compare the differences between each group. A *p*-value < 0.05 was considered statistically significant.

## Results

### Demographic data of the participants

This study enrolled 120 participants, including 93 men (77.5%) and 27 women, mainly aged ≥45 years (90%). The main proportion of the educational level was less than high school (35.8%). Forty-seven participants (39.2%) were diagnosed with lip and oral cavity cancer, 57 (47.5%) with nasopharyngeal, nasal, or laryngeal cancer, 12 (10%) with tonsil or parotid gland cancer, three (2.5%) with esophageal cancer, and one (0.8%) with bronchoalveolar cancer. Eighty-two participants (68%) had a DMFT score of > 12. In 59 participants (53%) were in group of “the years after radiation” less than or equal to 4 years, and in 53 participants (47%), were more than 4 years. Ninety-five participants (79.2%) had both substandard stimulated and unstimulated salivary flows, which met the criteria for xerostomia. We defined sleep disturbance as a PSQI score of more than 5 and xerostomia as a stimulated saliva flow rate of less than 0.5 mL/min. As shown in Table 1, 66 (55%) participants had both sleep disturbance and xerostomia. The prevalence of sleep disturbance and xerostomia comorbidities among head and neck cancer survivors was 55% (Table 1). The demographic data revealed that head and neck cancer tended to occur in men aged ≥45 years and in low to middle educational level groups. Major cancer diagnoses occurred at the oral and nasal sites and were associated with more dental problems. Cancer survivors continued to visit the dental clinic 4 years after radiotherapy. Cancer survivors commonly had a high DMFT index and poor salivary secretion after radiotherapy. Table 1 shows that the prevalence of xerostomia was 79.2%. These results indicate that some participants with dry mouth symptoms did not have sleep disturbances 46 (38.3%).]. Totally eight participants did not have dry mouth symptoms with sleep disturbance 3 (2.5%) and without sleep disturbance 5 (4.2%).

**Table 1:** Demographic data of the participants

Variables	Category	N	(%)
Sex			
	Male	93	(78)
	Female	27	(23)
Age			
	≤44	10	(8)
	45 ~ 64	78	(65)
	≥65	30	(25)
	Unknown	2	(2)
Education Level			
	Less than high school	43	(36)
	High school	42	(35)
	Collage or more	35	(29)
Major diagnosis			
	Lip and oral cavity cancer	47	(39)
	Nasopharyngeal or nasal Cavity or laryngeal cancer	57	(48)
	Tonsil or parotid gland cancer	12	(10)
	Esophageal cancer	3	(3)
	Bronchoalveolar cancer	1	(1)
DMFT			
	≤12	38	(32)
	> 12	82	(68)
Year after radiation			
	≤4	45	(38)
	> 4	60	(50)
Either USF or SSF	Substandard (< 0.1, < 0.5)	19	(14)
Both USF and SSF	Standard (≥ 0.1, ≥ 0.5)	6	(5)
	Substandard (< 0.1, < 0.5)	95	(79)
SSF ≥ 0.5	PSQI < 5	5	(4%)
	PSQI ≥ 5	3	(3%)
SSF < 0.5	PSQI < 5	46	(38%)
	PSQI ≥ 5	66	(55%)

*DMFT* Decayed, Missing, and Filled teeth index: *USF* unstimulated saliva flow rate (mL/min): *SSF* stimulated saliva flow rate (mL/min): missing data are excluded from this table

### Sleep disturbance and stimulated saliva flow rate were significantly improved in the fifth year after radiotherapy

We aimed to determine the distribution of sleep disturbance, sense of dry mouth, and salivary flow rate each year after radiotherapy. We measured sleep quality using the PSQI and dry mouth using the short-form xerostomia inventory and salivary flow rate. The average PSQI score, dry mouth score, unstimulated salivary flow rate, and stimulated salivary flow rate in the years following radiotherapy are shown in Table 2. The PSQI score and stimulated salivary flow rate improved by year. The Kruskal–Wallis test showed a significant improvement in the PSQI score (*p* = 0.03) and stimulated saliva flow rate (*p* = 0.04) compared to the first year and the fifth year (Fig. S1). Sleep quality was poorer in the first 3 years after radiotherapy. The dry mouth score was higher in the first year after radiotherapy. The unstimulated and stimulated saliva flow rates gradually improved; however, only the stimulated saliva flow rate significantly improved over time. The patterns of the PSQI, xerostomia scores, and saliva flow rate by year after radiotherapy are shown in Supplementary Fig. 1. The data revealed that sleep disturbances were ameliorated when the salivary flow rate improved. We detected both stimulated and unstimulated salivary flow rates. Only the stimulated salivary flow significantly improved over the years. The dry mouth score improved slightly over the years without statistical significance. Some participants felt fine with their dry mouth, which may have resulted in a low dry mouth score and substandard saliva flow simultaneously.

**Table 2:** The average PSQI score, xerostomia score, and salivary flow rate at different time periods (years) after radiotherapy (*n* = 105)

		PSQI score		Dry mouth score		USF (mL / min)		SSF (mL / min)	
YAR	N	Mean	SD	Mean	SD	Mean	SD	Mean	SD
1	8	9.25	4.46	12.25	2.12	0.03	0.03	0.11	0.10
2	13	7.15	4.38	11.08	3.52	0.03	0.02	0.06	0.08
3	12	9.25	4.90	11.25	4.52	0.05	0.06	0.12	0.13
4	12	7.42	4.72	11.17	3.66	0.05	0.06	0.18	0.19
5	12	5.58	4.03	11.00	2.80	0.09	0.10	0.21	0.16
> 5	48	5.22	3.24	10.27	3.13	0.07	0.10	0.20	0.18
*p*		0.03*		0.57		0.67		0.04*	

Kruskal–Wallis test; *p <  0.05(the first year v.s. the fifth year); Grouping variable: Year after radiotherapy (YAR); *USF* unstimulated saliva flow rate; *SSF* stimulated saliva flow rate; *SD* standard deviation; N = participant number

### Patients who had substandard salivary flow rates had higher DMFT scores

Saliva is associated with the maintenance of oral health. We aimed to determine whether the salivary flow rate affected oral health among cancer survivors. The DMFT was performed by a clinical dentist and recorded on the day the participants completed the questionnaire. The Mann−Whitney U test showed that participants with a substandard unstimulated saliva flow rate (< 0.1 mL/min) and stimulated saliva flow rate (< 0.5 mL/min) had higher DMFT mean rank. This indicated that head and neck cancer survivors with substandard saliva flow had a higher prevalence of dental caries (Table 3).

**Table 3:** Mean rank of DMFT among different salivary flow rate standard

	Variables	category	N	Mean Rank	Mann-Whitney U
DMFT	USF	≥0.1	23	49	851 *p* = 0.08
		< 0.1	97	63.23	
	SSF	≥0.5	8	49.13	357 *p* = 0.34
		< 0.5	112	61.31	

Mann−Whitney U test.

*SSF* stimulated salivary flow rate: *USF* unstimulated salivary flow rate: *DMFT* decayed, missing, and filled teeth.

### Linear regression analysis of the variables correlated to the sleep disturbance

We conducted linear regression to analyze the correlation between salivary flow rate, self-reported dry mouth score, and PSQI score (sleep disturbance). Both stimulated salivary flow rate and self-reported dry mouth score were significantly associated with PSQI score. The dry mouth score was significantly positively correlated with PSQI score (*p* = 0.03), while the stimulated salivary flow rate was significantly negatively correlated with PSQI score (*p* = 0.01) which did not affect by confounding factors (age and sex) (Table 4). These results indicated that when clinical dentists find that cancer survivors have a substandard stimulated salivary flow rate and high self-reported dry mouth score, they must consider the sleep disturbance that is comorbid with oral problems.

**Table 4:** Linear regression analysis of the variables correlated to the PSQI

	Unstandardized Coefficients		Standardized Coefficients	t	Sig.
	B	Std. Error	Beta		
(Constant)	2.71	2.63		1.03	0.30
U weight(g = ml/5 min)	11.25	5.55	0.21	2.03	0.05
S weight(g = ml/5 min)	−5.96	2.32	−0.27	−2.57	0.01*
Dry mouth score	0.24	0.11	0.19	2.15	0.03*
Age	0.04	0.04	0.08	0.92	0.36
Sex	−0.75	0.86	−0.08	−0.87	0.39

Dependent Variable: PSQI; *p < 0.05

## Discussion

This cross-sectional study explored the prevalence of comorbidities in sleep disturbance and xerostomia in the clinical end and analyzed the association of salivary flow rate and dry mouth score with sleep disturbance and the association of two different salivary flow rates (stimulated and unstimulated) with dental caries. Demographic data showed that men aged > 45 years old were more likely to suffer from head and neck cancer, and nearly 70% of the participants had an educational level at or below high school. Table 1 shows that 68% of the participants had a DMFT score of over 12. This provides evidence that radiotherapy-induced acute or chronic toxicity significantly affects the patient’s oral health. Both stimulated and unstimulated salivary flow rates were measured. Our data showed that the stimulated salivary flow rate and xerostomia score were significantly associated with sleep disturbance (Table 4).

However, xerostomia inventory is an subjective inventory that may affects by participants’ current mood. This was the reason why we detected the salivary flow which is an objective data as a indicator of dry mouth. We suggested that clinical dentists can use stimulated salivary flow rate as a predictor of oral health and sleep quality in cancer survivors.

Xerostomia is a typical symptom in patients who receive radiotherapy, affecting their quality of life. Researches demonstrated the patient quality of life after intensity-modulated radiotherapy (IMRT) was better than conventional radiotherapy. A meta-analysis also provided evidence that IMRT significantly improves xerostomia-related quality of life [15]. Currently, the head and neck cancer patients we have enrolled are receiving conventional radiotherapy. We suggested that oral surgeons consider IMRT for patients with head and neck cancer. Our data showed that the prevalence of xerostomia was nearly 80% among cancer survivors, and nearly 55% had sleep disturbance (Table 1). Medical professionals must recognize this high prevalence and try to help cancer survivors achieve a better quality of life during treatment. Sleep disturbance in head and neck cancer survivors starts from the pretreatment period owing to anxiety, a higher passive coping style, and oral pain [14, 16]. When a dentist manages the oral health problems of survivors of head and neck cancers, sleep problems should be noted and handled with the same priority. Dental professionals should be aware of this concept from when they are students. Sleep-related courses should be incorporated into the dental curriculum [17].

Quality of life has a significant impact 2–6 years after a cancer diagnosis [18]. Even after 12 months of treatment, patients still experience deterioration in physical function, fatigue, and xerostomia [19]. Based on our data, the salivary flow rate improved after 3 years of radiotherapy. Dental professionals may pay more attention and care more for head and neck cancer survivors within 3 years of treatment. The quality of life of head and neck cancer survivors is associated with the cancer stage and treatment methods. However, these data were not collected in this study. We aimed to identify a single marker that directly reflects the quality of life of patients with head and neck cancers after radiotherapy. Therefore, we focused on salivary flow rate and its association with oral health and sleep quality.

The strength of this study is that it analyzed the association between sleep disturbance and dry mouth using both subjective (Summated Xerostomia Inventory) and objective (salivary flow) measurements, elevating the reliability of study outcomes. However, this study has some limitations. This cross-sectional study could not predict the causal effect of salivary flow on DMFT or sleep disturbances. We enrolled participants at one dental clinic in the hospital; therefore, the research outcomes may not represent all head and neck cancer survivors who had received radiotherapy.

## Conclusions

This study showed that stimulated salivary flow was significantly associated with oral health and sleep quality in head and neck cancer survivors. Dental professionals should begin managing patients’ uncomfortable symptoms, in terms of both oral and mental health, before cancer treatment. This concept should be incorporated into the dental curricula.

### Supplementary Information

**Additional file 1.**

## Data Availability

No datasets were generated or analysed during the current study.

## Abbreviations

PSQI: Pittsburgh Sleep Quality Index
DMFT: Decayed, Missing, and Filled Teeth
USF: Unstimulated Saliva Flow Rate
SSF: Stimulated Saliva Flow Rate
