# Supplementary material for: The Association of Salivary Flow Rate and Sleep Quality among Head and Neck Cancer Survivors after Radiotherapy
Source: BMC Oral Health. 2024 Feb 19;24:251. doi: 10.1186/s12903-024-03977-5 (PMC10875849; doi:10.1186/s12903-024-03977-5)
Supplement: Supplementary file 1 — Additional file 1. [file 12903_2024_3977_MOESM1_ESM.docx]

**Supplementary Figure 1**. The average PSQI score, dry mouth score, and the salivary flow rate in each year after radiotherapy groups. The PSQI scores decreased and salivary flow increased with increasing years of therapy. *p < 0.05 Kruskal-Wallis Test compared to the first year and the fifth year.
